# Seroprevalence of SARS-CoV-2 IgG antibodies in children seeking medical care in Seattle, WA June 2020 to December 2022

**DOI:** 10.1128/spectrum.02625-24

**Published:** 2025-03-10

**Authors:** Amanda L. Adler, Alpana Waghmare, Kirsten Lacombe, Jane A. Dickerson, Alexander L. Greninger, Melissa Briggs Hagen, Kimberly Pringle, Tarayn Fairlie, Claire M. Midgely, Janet A. Englund

**Affiliations:** 1 Seattle Children’s Research Institute, Seattle, Washington, USA; 2 Department of Pediatrics, University of Washington 7284 , Seattle, Washington, USA; 3 Department of Laboratory Medicine and Pathology, University of Washington 7284 , Seattle, Washington, USA; 4 Coronavirus and Other Respiratory Viruses Division, Centers for Disease Control and Prevention 1242 , Atlanta, USA; Barnard College, Columbia University, New York, New York, USA

**Keywords:** pediatric, SARS-CoV-2, antibody, health disparities, serology

## Abstract

Seroprevalence studies play an important role in estimating the number of children infected with SARS-CoV-2. We report SARS-CoV-2 seroprevalence in children seeking medical care for any reason at a free-standing pediatric hospital in Seattle, WA over a 2.5-year period and four distinct pandemic waves. We randomly selected residual serum samples from children and young adults seeking medical care as inpatients and outpatients at Seattle Children’s Hospital between June 2020 and December 2022 to test for the presence of anti-nucleocapsid (N) antibodies. Samples were categorized into four distinct pandemic waves based on Washington State epidemiology: Wave 1 (June 2020–October 2020), Wave 2 (November 2020–June 2021), Wave 3 (July 2021–November 2021), and Wave 4 (December 2021–December 2022). Patient characteristics and COVID-19 vaccine status were obtained, and zip codes were used to ascertain the Social Vulnerability Index (SVI). Multivariable Poisson regression models with robust variance estimates were used to examine the relationship between patient characteristics and anti-N-positivity for each wave. Among 8,040 samples from 7,102 patients included in the analyses, seroprevalence rose from 2.4% (95% CI, 2.0%–3.1%) in Wave 1 to 25.5% (95% CI 23.3%–27.8%) in Wave 4 (following the Omicron surge). High SVI, Hispanic ethnicity, or use of government insurance was associated with increased anti-N positivity in most waves. We observed a steady increase in anti-N seroprevalence followed by a sharp increase after the Omicron surge in early 2022. Our data demonstrate the burden of COVID-19 on specific groups with health disparities within our region throughout the pandemic.

**IMPORTANCE:**

Our results highlight the importance of seropositivity studies as essential tools to provide information on the incidence and prevalence of SARS-CoV-2 seropositivity. Our results also reinforce other reports demonstrating the inequitable burden of COVID-19 on groups with health disparities and that this inequitable burden continued to persist throughout the pandemic, even in a region with high adherence to COVID-19 mitigation efforts. It also highlights SVI’s value in identifying communities that must be part of pandemic research, and public health and vaccination strategies.

## INTRODUCTION

SARS-CoV-2, the virus that causes COVID-19, was first detected in the United States in January 2020 and has caused over one million deaths in the United States (1). While children typically experience less severe COVID-19 than adults (2), infected children remain at risk for complications including hospitalization and multisystem inflammatory syndrome (3, 4).

Early data based on diagnosed and reported cases suggested children were less likely to be infected with SARS-CoV-2 than adults (5) but more recent studies demonstrate a similar prevalence of SARS-CoV-2 infection in children and adults (6, 7). However, children are more likely to have asymptomatic, mild, or atypical symptoms (8–10), which may result in missed infections and thereby underestimate the COVID-19 burden in this population (9). In addition, while data regarding health disparities with COVID-19 have been well documented in adults, there are few studies that have evaluated health disparities in relation to serological diagnosis of SARS-CoV-2 infection in children.

Antibodies produced following SARS-CoV-2 infection, including antibodies to spike (S) and nucleocapsid (N) proteins, have been used to estimate seroprevalence in multiple populations. Assays to these antibodies are sensitive, reliable, and have been licensed by regulatory authorities for clinical use, although assay characteristics are known to vary (11–13). Because anti-S antibody is detected following vaccination as well as infection, anti-N antibody is frequently utilized to detect infection in serosurveys (14–20). Surveillance studies have documented that N antibodies persist in adults, depending on the assay utilized, between 6 and 12 months post-infection (15, 16, 21) and up to and over 1 year in children (17, 18). Therefore, studies of SARS-CoV-2 anti-N can play an important role in estimating the number of children infected with SARS-CoV-2 as they can capture persistent antibodies developed by both asymptomatic and symptomatic cases and are unaffected by SARS-CoV-2 vaccination.

Here we report SARS-CoV-2 seroprevalence in children seeking medical care for any reason at a free-standing pediatric hospital in Seattle, WA over a 2.5-year period and four distinct pandemic waves.

## MATERIALS AND METHODS

### Setting and institutional review

Seattle Children’s Hospital (SCH) is a 407-bed, quaternary-care pediatric hospital serving Washington State and the Pacific Northwest, with over 390,000 patient visits annually including admissions to the hospital and ambulatory visits.

### Sample collection

We collected residual sera samples from children seeking medical care as inpatients and outpatients, for any reason, at SCH between June 2020 and December 2022. We randomly selected residual sera monthly from roughly equal numbers of subjects in four age groups (<5 years, 5 to <10 years, 10 to <15 years, and 15 years or older). The number of samples selected each month varied between 100 and 800 over the course of the study (Fig. S1). We aimed to select an equal proportion of samples from both hospitalized and outpatient children within each age group per month, as samples were available. Samples from patients on the hematology/oncology or hematopoietic stem cell inpatient service or visiting these outpatient clinics were excluded due to the overrepresentation of samples collected via frequent daily blood collection from this population. We did not exclude samples from children with other immunocompromising conditions or oncology patients with blood samples if collected from other hospital locations (e.g., PICU).

### Data collection

Demographics, clinical characteristics of the encounter during which the sample was collected (Table 1), ICD-10 discharge codes, and insurance status were extracted from the electronic medical record. Data regarding receipt of SARS-CoV-2 vaccination were extracted from the Washington State Immunization Information System (WAIIS), which is highly accurate and complete (22), or the medical record (for those who live outside of Washington state).

**TABLE 1:** Seroprevalence among demographics and characteristics overall and by pandemic wave^*a*^

	Wave 1 (Jun 2020–Oct 2020) Positives/total % positivity (95% CI)	Wave 2 (Nov 2020–June 2021) Positives/total % positivity (95% CI)	Wave 3 (Jul 2021–Nov 2021) Positives/total % positivity (95% CI)	Wave 4 (Dec 2021–Dec 2022) Positives/total % positivity (95% CI)	Overall positives/total % positivity (95% CI)
Total samples	70/2821 2.5% (2.0%–3.1%)	158/2866 5.5% (4.7%–6.4%)	66/867 7.6% (6.0%–9.6%)	379/1486 25.5% (23.3%–27.8%)	673/8040 8.4% (7.8%–9.0%)
Age group <5 years 5–10 years 11–14 years >15 years	18/691 2.6% (1.6%–4.1%) 16/718 2.2% (1.4%–3.6%) 20/706 2.8% (1.8–4.4%) 16/706 2.3% (1.4–3.7%)	31/638 4.9% (3.4%–6.8%) 33/762 4.3% (3.1%–6.0%) 56/748 7.5% (5.8%–9.6%) 38/680 5.3% (3.9%–7.2%)	17/202 8.4% (5.3%–13.1%) 15/231 6.5% (3.9%–10.5%) 14/209 6.7% (4.1%–10.0%) 20/225 8.9% (5.8%–13.4%)	81/352 23.0% (18.9%–27.7%) 102/393 26.0% (21.9%–30.5%) 103/359 28.7% (24.2%–33.6%) 93/382 24.3% (20.3%–28.9%)	147/1883 7.8% (6.7%–9.1%) 166/2104 7.9% (6.8%–9.1%) 193/2022 9.6% (8.3%–10.9%) 167/2031 8.2% (7.1%–9.5%)
Sex Male Female	30/1367 2.2% (1.5–3.1%) 40/1454 2.8% (2.0%–3.7%)	64/1312 4.9% (3.8%–6.2%) 94/1554 6.0% (5.0%–7.3%)	30/427 7.0% (5.0%–9.9%) 36/440 8.2% (6.0%–11.1%)	173/694 24.9% (21.8%–28.2%) 206/791 26.0% (23.1%–28.3%)	297/3800 7.8% (7.0%–8.7%) 376/4239 8.9% (8.1%–9.7%)
Race White Black Other Unknown	47/1868 2.5% (1.9%–3.3%) 8/185 4.3% (2.1%–8.4%) 15/768 2.0% (1.2%–3.2%) None	108/1840 5.9% (4.9%–7.0%) 12/208 5.8% (3.3%–9.9%) 38/815 4.7% (3.4%–6.3%) 0/3 (0%)	47/568 8.3% (6.3%–10.8%) 5/51 9.8% (4.1%–21.6%) 14/245 5.7% (3.4%–9.4%) 0/3 (0%)	255/958 26.6% (23.95%–29.5%) **38/108** **35.2% (26.7%–44.6%**) 85/419 20.29% (16.7%–24.4%) 1/1 (100%)	457/5234 8.7% (8.0%–9.5%) 63/552 11.4% (9.0%–14.3%) 152/2247 6.7% (6.0%–7.9%) 1/7 (14%)
Ethnicity Hispanic Non-Hispanic Unknown	**36/526** **6.8% (5.0%–9.3%**) 31/2164 1.4% (1.0%–2.0%) 3/131 2.3% (0.7%–6.9%)	**72/579** **12.4% (10.0%–15.4%**) 83/2142 3.9% (3.1%–4.8%) 3/145 2.1% (0.7%–6.2%)	**22/172** **12.8% (8.5%–18.7%**) 41/645 6.4% (4.7%–8.5%) 3/50 6.0% (1.9%–17.2%)	**94/307** **30.6% (25.7%–36.0%**) 271/1110 24.4% (22.0%–27.0%) 14/69 20.3% (12.3%–31.5%)	**224/1584** **14.1% (12.5%–15.9%**) 426/6061 7.0% (6.4%–7.7%) 23/395 5.8% (3.9%–8.6%)
Location Hospitalized Outpatient	35/1112 3.2% (2.3%–4.4%) 35/1709 2.0% (1.5%–2.8%)	63/942 6.7% (5.3%–8.5%) 95/1924 4.9% (4.3%–6.4%)	30/309 8.9% (6.3%–12.4%) 36/528 6.8% (5.0%–9.3%)	132/519 25.4% (21.8%–28.4%) 247/967 25.5% (22.9%–28.4%)	260/2912 8.9% (7.9%–10.0%) 413/5128 8.1% (7.3%–8.8%)
Insurance Government Private None Unknown	**50/1283** **3.9% (2.9%–5.1%**) 10/1158 0.9% (0.4%–1.6%) 0/16 0% 10/364 2.8% (1.3%–5.0%)	**109/1515** **7.2% (5.9%–8.6%**) 47/1309 3.6% (2.6%–4.7%) 2/20 10% (1.2%–31.7%) 0/22 0%	**50/504** **9.92% (7.5%–12.9%**) 14/352 4.0% (2.2%–6.6%) 2/10 20% (2.5%–55.6%) 0/1 0%	**257/866** **29.7% (26.6%–32.8%**) 119/609 19.5% (16.4%–22.9%) 3/11 27.3% (6.0%–61.0%) None	**466/4168** **11.2% (10.3%–12.1%**) 190/3428 5.5% (4.8%–6.4%) 7/57 12.2% (5.9%–23.7%) 10/387 2.6% (1.4%–4.7%)
SVI level Low Low-medium Medium-high High Unavailable	7/494 1.4% (0.06%–2.9%) 15/1124 1.3% (0.07%–2.2%) 28/896 3.1% (2.1%–4.5%) **18/285** **6.3% (3.8%–9.8%**) 2/22 9.1% (1.1%–29.2%)	13/496 2.6% (1.4%–4.4%) 44/1108 4.0% (2.9%–5.3%) 69/935 7.4% (5.8%–9.3%) **32/300** **10.7% (7.4%–14.7%**) 0/27 0%	9/152 5.9% (2.7%–10.9%) 25/327 7.7% (5.0%–11.1%) 23/296 7.8% (5.0%–11.4%) 9/84 10.7% (5.0%–19.4%) 0/8 0%	41/223 18.4% (13.5%–24.1%) 140/602 23.3% (19.9%–26.8%) 137/481 28.5% (24.5%–32.7%) **56/164** **34.2% (26.9%–41.9%**) 5/16 31.1% (11.0%–58.7%)	70/1365 5.1% (4.1%–6.4%) 224/3161 7.1% (6.2%–8.0%) 257/2608 9.8% (8.8%–11.1%) **115/833** **13.8% (11.6%–16.3%**) **7/73** 9.6% (4.6%–19.1%)
Received any COVID-19 vaccine prior to the sample Yes No Not applicable^*b*^	None None 70/2821 2.5% (1.9%–3.1%)	7/132 5.3% (2.2%–10.6%) 18/185 9.7% (5.9%–14.9%) 133/2549 5.2% (4.4%–6.2%)	9/234 3.9% (1.8%–7.2%) **28/205** **13.7% (9.3%–19.1%**) 29/428 6.8% (4.6%–9.6%)	154/712 21.6% (18.7%–24.8%) **169/495** **34.1% (30.0%–38.5%**) 56/279 20.1% (15.5%–25.3%)	170/1078 15.8% (13.7%–18.1%) **215/885** **24.3% (21.6%–27.2%**) 288/6077 4.7% (4.2%–5.3%)

^*a*^ Characteristics in bold represent those with a statistical increase in seroprevalence (*P*<0.05; X^2^ or Fisher’s exact test).

^*b*^ COVID vaccine not yet available for not yet available for patient age.

Patient zip codes were used to ascertain the Social Vulnerability Index (SVI). Developed by the Centers for Disease Control and Prevention, the SVI provides a composite measure of community susceptibility to disasters, including disease outbreaks (23). It encompasses four subindices derived from American Community Survey data (2014–2018) focusing on socioeconomic status, household composition, disability, racial/ethnic minority status and language, housing type, and transportation. The cumulative SVI is determined by aggregating individual indices and transforming the resulting score into a percentile rank, ranging from 0 to 1, with higher values indicating increased vulnerability.

### Definitions

Samples were collected across four distinct pandemic waves. The initial pandemic wave (Wave 1) was defined as the start of the study period (June 2020) through October 2020; Wave 2 was defined as November 2020 through June 2021, during which the alpha variant was first detected and became the dominant variant in our region; Wave 3 was defined as July 2021 through November 2021 during which the delta variant was detected and became the dominant variant in our region, and Wave 4 was defined as December 2021 through December 2022, during which the omicron variant was detected and became the dominant variant in our region (24, 25).

Underlying medical conditions were categorized using ICD-10 codes grouped using the Complex Chronic Condition categories previously described by Feudtner et al., (26) with the addition of groups for asthma (ICD-10 codes J45-J45.998), diabetes (ICD-10 codes E08-E13.9), and obesity/pediatric BMI >95% (ICD-10 codes E66-E66.9 and Z68.54). SVI was categorized as low (0- < 0.25), low-medium (0.25–0.49), medium-high (0.5–0.74), and high (>=0.75) (27).

### Laboratory testing

All serum samples were tested for antibodies against the SARS-CoV-2 N protein by a chemiluminescent microparticle immunoassay (CMIA) using the Abbott Architect system. The Architect assay is a licensed qualitative test that detects IgG antibodies against the SARS-CoV-2 N protein. The manufacturer reports an assay sensitivity of 100% and specificity of 99.6% (28). The system calculates a calibrator mean chemiluminescent signal, and the default result unit is index (S/C). Index values greater than 1.4 were considered positive per the manufacturer’s instructions (12).

### Neutralization assays

All samples with anti-N antibodies detected, gathered between June 2020 and January 2022, underwent neutralization assays as previously described (29). Results are presented as the 50% neutralizing dilution titers (ND50).

### Statistical analyses

In all analyses, we included each subject’s initial specimen per pandemic wave. Since we assessed antibodies against the SARS-CoV-2 N protein, a measure unaffected by SARS-CoV-2 vaccination (20), vaccinated children were included in both bivariate and multivariable analyses. However, they were excluded from the analysis of neutralization assay data to ensure this analysis reflected infection alone. Furthermore, vaccination status at sample collection was not evaluated in the multivariable model as it does not represent vaccination status at the time of acute infection.

SARS-CoV-2 anti-N antibody positivity among children seeking medical care was reported by pandemic wave overall and by age group to account for the age-stratified sample collection, with 95% confidence intervals obtained using exact binomial distributions. The significance of the trend of SARS-CoV-2 antibody positivity was evaluated using the Cochran-Armitage trend test. Washington state census data were used to compare the distribution of demographics of the study cohort to that of children in Washington state (30). ND50 results were log-transformed and summarized by a wave.

We first examined the relationship between demographic and clinical characteristics and SARS-CoV-2 antibody positivity within each wave using either the Chi-squared test or Fisher’s exact test, as appropriate. Next, we conducted a multivariable analysis using Poisson regression with robust variance. Covariates of interest included those with a *P*-value of ≤0.1 in bivariate analyses for each wave. Variables that were not statistically significant (*P* < 0.05) were removed from the final multivariable model. Interaction with age group was assessed for each predictor evaluated in the model to account for the age-stratified sample collection. All analyses were repeated using the last specimen per patient per pandemic wave to ensure there were no significant differences in the results. The software package, R version 4.2.3 (The R Foundation for Statistical Computing) was used for all analyses.

## RESULTS

During the study period, 8,040 remnant sera samples were collected from 7,102 unique patients. Of these, the median age of the cohort was 11 years (IQR 5.3–15.0 years; range 0–27 years), 52.7% were female, 65.1% were white, and 19.7% were Hispanic. The vast majority (93%) of the cohort resided in Washington state; samples were collected from patients residing in 34 of Washington’s 39 counties (Fig. S2). The distribution of zip codes within our cohort matched that of our overall hospital population. Metabolic conditions, which included obesity and diabetes, were the most common health condition category among our subjects (17.9%), with obesity being the most common condition within that category (31.8%).

**Fig 1:**
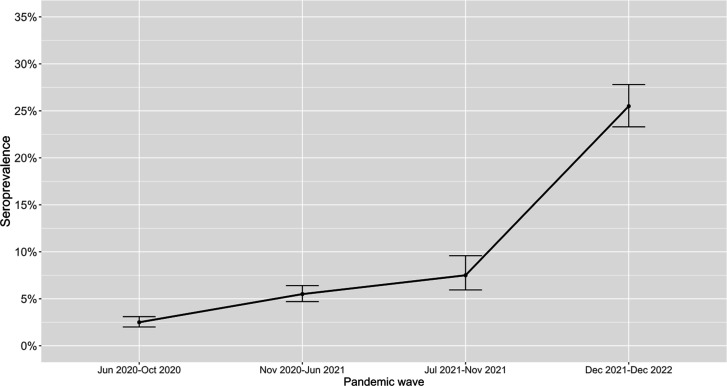
Seroprevalence by wave. Error bars indicate 95% confidence intervals.

The raw antibody positivity rate for Wave 1 was 2.4% (95% CI, 2.0%–3.1%), which increased to 5.5% (95% CI, 4.7%–6.4%) in Wave 2, 7.6% (95% CI, 6.0%–9.6%) in Wave 3, and 25.5% (95% CI 23.3%–27.8%) in Wave 4 (*P* value for overall trend <0.001; Fig. 1). The results were similar when stratified by age group (Table 1; Fig. S3). The distribution of demographics within the study cohort matched that of Washington State (data not shown); therefore, seroprevalence rates were not adjusted for demographic factors.

**Fig 2:**
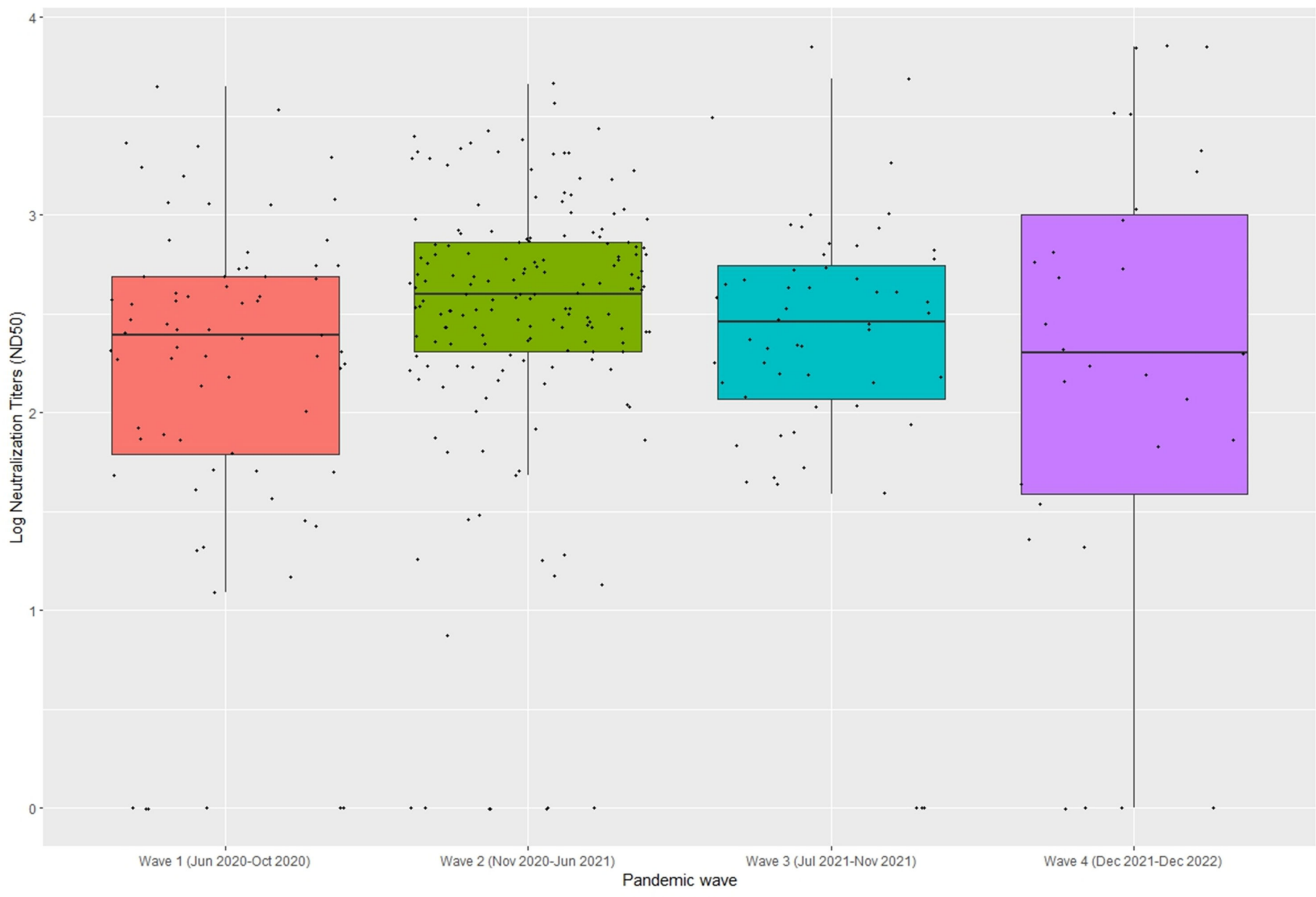
Distribution of ND50 titers by wave.

Altogether, ND50 data were available for 305 N-antibody-positive samples collected from unvaccinated children in Waves 1–3 and the first 6 weeks of Wave 4. Of the 305 samples tested, neutralizing antibodies were detected in 276 (90%). Of the 29 N-antibody positive samples that did not have neutralizing antibodies detected, 15 (52%) had an index value that was close to the level of positivity (average = 1.6) and 4 (14%) had a high index value (average = 6.0). The distribution of ND50 titers by wave is reported in Fig. 2 and did not differ by wave.

The demographic characteristics and percent positivity for anti-N antibodies by wave are shown in Table 1. In all waves, unadjusted seropositivity was higher in children of Hispanic ethnicity or those with government insurance. Similarly, seropositivity was higher in children residing in a zip code with a high SVI in all waves except Wave 3. Seroprevalence was higher among Black children than white children or children from “other” races (those not identifying as white or Black) in Waves 1, 3, and 4, but this difference was only statistically significant in Wave 4. No single race within the “other” racial category (Asian; American Indian or Alaska Native; or Native Hawaiian or other Pacific Islander) was sufficiently represented for further analysis.

During the study, 1,963 children were eligible for COVID-19 vaccination at the time of sample collection. Of those, 1,078 (55%) had received at least 1 dose of vaccine. In Waves 3 and 4, anti-N antibody positivity was statistically significantly higher among vaccine-eligible unvaccinated children than those who had received at least one dose.

Table 2 presents chronic condition categories by wave. Unadjusted seropositivity was higher among children with a metabolic condition, compared to those without a metabolic condition, in Waves 2 and 3 only. No other chronic condition categories were associated with increased seroprevalence.

**TABLE 2:** Seroprevalence among underlying medical condition categories^a^

	Wave 1 (Jun 2020–Oct 2020) Positives/total % positivity (95% CI)	Wave 2 (Nov 2020–June 2021) Positives/total % positivity (95% CI)	Wave 3 (Jul 2021–Nov 2021) Positives/total % positivity (95% CI)	Wave 4 (Dec 2022–Dec 2022) Positives/total % positivity (95% CI)	Overall positives/total % positivity (95% CI)
Any condition Yes No	34/1,492 2.3% (1.6%–3.2%) 36/1,329 2.7% (2.0%–3.7%)	83/1,440 5.8% (4.7%–7.1%) 75/1,426 5.3% (4.2%–6.5%)	36/482 7.5% (5.4%–10.2%) 30/385 7.8% (5.5%–10.9%)	179/722 24.8% (21.7%–28.1%) 171/662 25.8% (22.5%–29.3%)	332/4,136 8.0% (7.2%–8.9%) 312/3802 8.2% (7.4%–9.1%)
Neuromuscular Yes No	5/240 2.1% (0.87–4.9%) 65/2581 2.5% (2.0%–3.2%)	4/210 1.9% (0.7%–4.8%) 154/2656 5.8% (5.0%–6.8%)	4/77 5.2% (1.9%–13.1%) 62/790 7.9% (6.1%–9.9%)	28/110 25.4% (17.6%–34.6%) 322/1274 25.3% (22.9%–27.8%)	41/637 6.4% (4.7%–8.6%) 603/7301 8.3% (7.6%–8.9%)
Cardiovascular Yes No	6/338 1.8% (0.8%–3.9%) 64/2483 2.6% (2.0%–3.3%)	13/262 5.0% (2.9%–8.4%) 145/2604 5.6% (4.7%–6.5%)	6/114 5.3% (2.4%–11.3%) 60/753 8.0% (6.2%–10.1%)	52/185 28.1% (21.7%–35.2%) 298/1199 24.8% (22.4%–27.4%)	77/899 8.6% (6.8%–10.6%) 567/7039 8.1% (7.4%–8.7%)
Respiratory +asthma Yes No	1/135 0.74% (0.1%–5.1%) 69/2686 2.6% (2.0%–3.2%)	5/127 3.9% (1.6%–9.1%) 153/2739 5.6% (4.8%–6.5%)	2/59 3.4% (0.08%–12.7%) 64/808 7.9% (6.2%–10.0%)	21/95 22.1% (14.2%–31.7%) 329/1289 25.5% (23.2%–28.0%)	29/416 7.0% (4.7%–9.9%) 615/7522 8.2% (7.6%–8.8%)
Renal Yes No	7/210 3.3% (1.6%–6.8%) 63/2611 2.4% (1.9%–3.1%)	8/191 4.2% (2.1%–8.2%) 150/2675 5.6% (5.0%–6.5%)	7/77 9.1% (4.4%–18.0%) 59/790 7.5% (5.8%–9.5%)	24/94 25.5% (17.1%–35.6%) 326/1290 25.3% (22.9%–27.7%)	46/572 8.0% (5.9%–10.6%) 598/7366 8.1% (7.5%–8.8%)
Gastrointestinal Yes No	6/391 1.5% (0.7%–3.4%) 64/2430 2.6% (2.1%–3.4%)	18/303 5.9% (3.8%–9.2%) 140/2563 5.5% (4.6%–6.4%)	9/129 7.0% (3.7%–12.9%) 57/738 7.7% (6.0%–9.9%)	36/178 20.2% (14.6%–26.9%) 314/1206 26.0% (23.6%–28.6%)	69/1001 6.9% (5.4%–8.6%) 575/6937 8.3% (7.7%–9.0%)
Hematologic Yes No	2/144 1.4% (0.35%–5.4%) 68/2677 2.5% (2.0%–3.2%)	8/123 6.5% (3.3%–12.5%) 150/2743 5.5% (4.7%–6.4%)	5/40 12.5% (5.2%–27.0%) 61/827 7.4% (5.8%–9.4%)	23/78 29.5% (19.7%–40.9%) 327/1306 25.0% (22.7%–27.5%)	38/385 9.9% (7.1%–13.3%) 606/7553 8.0% (7.4%–8.7%)
Metabolic +diabetes + obesity Yes No	15/458 3.3% (2.0%–3.0%) 55/2363 2.3% (1.8%–3.0%)	**47/553** **8.5% (6.4%–11.3%**) 111/2313 4.8% (4.0%–5.7%)	**24/165** **14.5% (9.9%–20.8%**) 42/702 6.0% (4.4%–8.0%)	61/247 24.7% (19.4%–30.6%) 289/1137 25.4% (22.9%–28.1%)	147/1423 10.3% (8.8%–12.1%) 497/6515 7.6% (7.0%–8.3%)
Congenital Yes No	2/155 1.3% (0.03%–5.0%) 68/2666 2.5% (2.0%–3.2%)	2/146 1.4% (0.03%–5.3%) 156/2720 5.7% (4.9%–6.8%)	2/48 4.2% (1.0%–15.4%) 64/819 7.8% (6.2%–9.9%)	16/68 23.5% (14.1%–35.4%) 334/1316 25.4% (23.0%–27.9%)	22/417 5.3% (3.3%–7.9%) 622/7521 8.3% (7.7%–8.9%)
Malignancy Yes No	4/169 2.4% (0.09%–6.2%) 66/2652 2.5% (1.9%–3.2%)	6/162 3.7% (1.7%–8.0%) 152/2704 5.6% (4.8%–6.6%)	3/49 6.1% (2.0%–17.5%) 63/818 7.7% (6.1%–9.7%)	17/52 32.7% (20.3%–47.1%) 333/1332 25.0% (22.7%–27.4%)	30/432 6.9% (4.7%–9.8%) 614/7506 8.2% (7.6%–8.8%)
Neonatal Yes No	2/35 5.7% (1.4%–20.5%) 68/2786 2.4% (1.9%–3.1%)	1/28 3.6% (0.05%–22.1%) 157/2838 5.5% (4.7%–6.4%)	0/10 0.0% 66/857 7.7% (6.1%–9.7%)	9/32 28.1% (13.7%–46.7%) 341/1352 25.2% (22.9%–27.6%)	12/105 11.4% (6.0%–19.1%) 632/7833 8.1% (7.5%–8.7%)
Devices Yes No	11/369 3.0% (1.7%–5.3%) 59/2452 2.4% (1.9%–3.1%)	11/282 3.9% (2.2%–6.9%) 147/2584 5.7% (4.8%–6.7%)	7/115 6.0% (2.9%–12.3%) 59/752 7.8% (6.1%–10.0%)	45/181 24.9% (18.7%–31.8%) 305/1203 25.4% (22.9%–27.9%)	74/947 7.8% (6.2%–9.7%) 570/6991 8.2% (7.5%–8.8%)
Transplant Yes No	3/51 5.9% (1.9%–16.9%) 67/2770 2.4% (1.9%–3.1%)	6/34 17.7% (8.0%–34.4%) 152/2832 5.4% (5.0%–6.3%)	2/7 28.7% (6.3%–70.2%) 64/860 7.4% (5.9%–9.4%)	7/16 43.8% (19.7%–70.1%) 343/1368 25.1% (22.7%–27.5%)	18/108 16.7% (10.2%–25.1%) 626/7830 8.0% (7.4%–8.6%)
Number of chronic condition categories None 1 category ≥2 categories	36/1329 2.7% (1.8%–3.5%) 16/907 1.8% (1.4%–3.4%) 18/585 3.1% (1.6%–4.5%)	75/1426 5.3% (3.5%–7.5%) 56/891 6.3% (5.0%–8.3%) 27/549 4.9% (3.5%–7.5%)	30/385 7.8% (5.5%–10.7%) 18/269 6.7% (4.2%–10.4%) 18/213 8.5% (5.5%–13.6%)	200/764 26.2% (22.8%–29.0%) 100/419 23.8% (21.2%–29.4%) 79/303 26.1% (20.7%–30.7%)	341/3904 8.7% (7.9%–9.7%) 190/2486 7.6% (6.6%–8.8%) 142/1650 8.6% (7.3%–10.1%)

^*a*^ Characteristics in bold represent those with a statistical increase in seroprevalence (*P* < 0.05; X^2^ or Fisher’s exact test).

In multivariable analysis, no single characteristic was associated with a higher seropositivity in all waves. In Waves 1–3, anti-N seropositivity was higher in Hispanic children compared to children of non-Hispanic ethnicity, but not in Wave 4 (Table 3). Similarly, seropositivity was higher in children with government insurance than those with private insurance in Waves 1, 3, and 4. Furthermore, children who resided in a zip code with a high SVI had higher seropositivity in Waves 2 and 4, and children with a metabolic chronic condition had higher seropositivity in Wave 3 (Table 3). Age group interaction terms were not statistically significant and were removed from the final models.

**TABLE 3:** Bivariate and multivariable analyses of potential factors associated with seroprevalence and results reported as prevalence ratio and 95% CI

	Wave 1		Wave 2		Wave 3		Wave 4	
	Unadjusted	Adjusted	Unadjusted	Adjusted	Unadjusted	Adjusted	Unadjusted	Adjusted
Race White Black Other	Reference 1.72 (0.82–3.58)^*a*^ 0.78 (0.44–1.40)^*a*^	^*b*^	Reference 0.98 (0.55–1.75)^*a*^ 0.79 (0.55–1.14)^*a*^	^*b*^	Reference 1.18 (0.49–2.85)^*a*^ 0.69 (0.39–1.23)^*a*^	^*b*^	Reference 1.32 (1.00–1.74) 0.76 (0.61–0.95)	^*b*^
Hispanic ethnicity	4.77 (2.98–7.65)	3.28 (1.98–5.44)	3.21 (2.37–4.34)	2.84 (2.04–3.94)	2.01 (1.23–3.28)	1.67 (1.01–2.77)	1.25 (1.03–1.53)	^*b*^
Government insurance^*c*^	4.51 (2.29–8.85)	3.48 (1.70–7.09)	2.00 (1.43–2.80)	^*b*^	2.49 (1.40–4.44)	1.87 (1.02–3.43)	1.52 (1.25–1.83)	1.44 (1.19–1.76)
High SVI category^*d*^	3.17 (1.88–5.37)	^*b*^	2.14 (1.49–3.11)	1.59 (1.07–3.37)	1.46 (0.75–2.83)^*a*^	^*b*^	1.40 (1.11–1.77)	1.29 (1.02–1.64)
Metabolic condition^*e*^	1.41 (0.82–2.47)^*a*^	^*b*^	1.77 (1.27–2.46)	^*b*^	2.43 (1.51–3.90)	2.28 (1.40–3.70)	0.96 (0.76–1.22)^*a*^	^*b*^

^*a*^ *P* value >0.1.

^*b*^ Did not meet criteria for inclusion in the final model.

^*c*^ Evaluated as government vs private insurance.

^*d*^ Evaluated as high SVI vs other SVI levels.

^*e*^ Defined as diabetes, obesity, or other conditions meeting the definition of metabolic conditions by Feudtner et al. (26).

## DISCUSSION

We performed a cross-sectional seroprevalence study using residual clinical samples from children seeking medical care at a single regional institution over a 2.5-year period and four distinct pandemic waves. We observed a steady increase in anti-N seroprevalence followed by a sharp increase after the Omicron surge in early 2022 (Wave 4). Of note, based on the detection of anti-N antibodies, we documented that seroprevalence rates increased from <3% during the beginning of the COVID-19 pandemic to nearly one in three subjects during the Omicron period (which included Omicron sub-variants BA.1, BA.2, and BA.4/5). We also found that Hispanic ethnicity, government insurance use, high SVI, and underlying metabolic conditions were associated with higher seroprevalence, but specific characteristics differed by wave.

It is challenging to compare our data to other reports as we defined pandemic waves based on regional epidemiology, which does not directly overlap with time periods or methods used to define epidemiology in other studies, in part because of the extensive viral testing and surveillance that was conducted in our region (24, 25). In addition, WA State adhered widely to state-recommended social distancing guidelines, including masking (31). In general, we observed lower seroprevalence rates than other published pediatric data (32–34). Boehme et al. tested remnant serum samples collected from children seeking healthcare in Arkansas for SARS-CoV-2 antibodies and reported seroprevalence rates of 9.5%–23.4% between June 2020 and April 2021 (32), whereas our observed rates ranged from 2.5% to 7.6% during a similar period. Furthermore, the Centers for Disease Control and Prevention Multistate Assessment of SARS-CoV-2 Seroprevalence in Commercial Labs (MASS-C) estimates of pediatric seroprevalence in Washington were much higher than our observed rates (33). These differences in our observed rates versus others may be due to differences in the study population, in methodology for estimating seroprevalence, or differences in adherence to mask mandates, duration of school closures, and/or the assay used. For instance, a report of seroprevalence among children seeking medical care in Oregon yielded results very similar to our study (3.2%–30% vs 2.5%–25.5% during a similar period) (34). The cohort of children seeking medical care in Oregon may be more similar to that of Washington than cohorts in other regions of the country, and adherence to state mandates in Oregon may have been more similar to those in Washington State than in other parts of the US.

Neutralizing activity was documented in 91% of specimens testing positive for anti-N antibodies, suggesting that seropositivity was associated with some level of immune protection. While the Abbott Architect anti-N chemiluminescent assay used has high sensitivity in the weeks to months immediately after infection, this assay preferentially detects low-affinity antibodies which are known to wane over time, potentially even more than in other anti-N antibody assays (35, 36). Therefore, this assay may fail to detect individuals infected with SARS-CoV-2 whose anti-N IgG response waned below the positive threshold prior to sample collection. When evaluating the 9% of sera that were not concordant, most samples had either a very low index value (i.e., a very low positive N-antibody value) or a very high index value indicating the remnant sample was likely collected very close to the acute infection.

Ethnic minority groups and other populations with health disparities have been well documented to be disproportionately affected by the COVID-19 pandemic, which likely reflects multiple socioeconomic inequities including economic instability and work circumstances (37, 38). We found that children with government insurance were more likely to have SARS-CoV-2 antibodies compared to children with private insurance in Waves 1, 3, and 4. Similarly, children with Hispanic ethnicity were more likely to have SARS-CoV-2 antibodies compared with non-Hispanic children in Waves 1–3, but not Wave 4. It is possible that Omicron’s enhanced transmissibility is responsible for the similar infection rates between ethnic groups seen in Wave 4. Residing in a zip code with a high social vulnerability index was associated with increased seroprevalence in Waves 2 and 4, a finding which did persist despite Omicron’s enhanced transmissibility. Our findings are consistent with other reports that identified increased seroprevalence among children with Hispanic ethnicity (32, 39, 40) and those belonging to households with lower socioeconomic status (40, 41). Similarly, our finding of increased seroprevalence among children residing in geographic areas with a high level of social vulnerability is also consistent with previous reports (42, 43). Escobi et al. reported that counties in South Carolina with high SARS-CoV-2 seroprevalence were also the counties with a high SVI category (42). Importantly, more recent studies report lower pediatric SARS-CoV-2 vaccination in areas of high social vulnerability (44, 45). Even though no single characteristic was associated with seroprevalence in all waves, our data, taken together, further demonstrate that the pandemic exacerbated known health disparities.

This study has several strengths, including its large cohort of children and prolonged study period across several pandemic waves using an FDA-authorized antibody assay, and evaluation of SVI as a predictor for seroprevalence but also some notable limitations. First, some race groups are underrepresented in the study. While this is reflective of the demographics of Washington State, we were unable to fully assess race as a risk factor for seroprevalence. We note that, as with other seroprevalence studies, we are unable to assess the timing of infection in our assessment. We also acknowledge that Wave 4 consisted of Omicron sub-variants BA.1, BA.2, BA.4/5 and lacked enough data to evaluate these sub-variants as separate waves; however, we feel it is reasonable to treat these sub-variants as a single wave as all three variants share greater similarities than with pre-Omicron variants. Furthermore, this report is limited by its use of convenience sampling, which may reduce generalizability. We included each subject’s initial specimen per pandemic wave which may have allowed persistently positive antibodies from a prior wave to be counted again in the next “wave”; however, this is unlikely to have impacted our results as only 15 positive samples (2%) were collected from a subject who had contributed a positive sample to a previous wave, and the majority of sera were collected >6 months apart, with 25% > 12 months apart (data not shown). Finally, the study included clinically obtained samples collected from children seeking medical care; therefore, our report may overrepresent children with greater healthcare access or a greater need for healthcare based on underlying conditions.

Our results highlight the importance of seropositivity studies as essential tools to provide information on the incidence and prevalence of SARS-CoV-2 seropositivity. Our results also reinforce other reports demonstrating the inequitable burden of COVID-19 on groups with health disparities and that this inequitable burden continued to persist throughout the pandemic. It also highlights SVI’s value in identifying communities that must be part of pandemic researchand public health and vaccination strategies.

## Data Availability

The data are not publicly available due to privacy or ethical restrictions. The data that support the findings of this study are available from the corresponding author upon reasonable request.

## SUPPLEMENTAL MATERIAL

Fig S1 spectrum.02625-24-s0001.pdf Sample collection strategy.

Fig S2 spectrum.02625-24-s0002.tif Seroprevalence by Washington State County by Wave.

Fig S3 spectrum.02625-24-s0003.tif Seroprevalence by wave stratified by age group.

Fig S4 spectrum.02625-24-s0004.pdf Proportion of positive samples and new case counts by month and wave.

Supplemental figure legends spectrum.02625-24-s0005.docx Proportion of positive samples and new case counts by month and wave

ASM does not own the copyrights to Supplemental Material that may be linked to, or accessed through, an article. The authors have granted ASM a non-exclusive, world-wide license to publish the Supplemental Material files. Please contact the corresponding author directly for reuse.
