# Supplementary material for: Seroprevalence of SARS-CoV-2 IgG antibodies in children seeking medical care in Seattle, WA June 2020 to December 2022
Source: Microbiol Spectr. 2025 Mar 10;13(4):e02625-24. doi: 10.1128/spectrum.02625-24 (PMC11960482; doi:10.1128/spectrum.02625-24)

Age Group	# Samples included/ # Collected	# Samples included/ # Collected	# Samples included/ # Collected	# Samples included/ # Collected	Total samples included/Total collected
0-<5 Years	604/807	692/1004	489/613	98/125	1883/2549
5-10 Years	624/801	816/990	549/614	115/136	2104/2541
11-14 Years	618/783	797/1002	497/563	110/122	2022/2470
>= 15 Years	620/806	768/1004	535/606	108/129	2031/2545

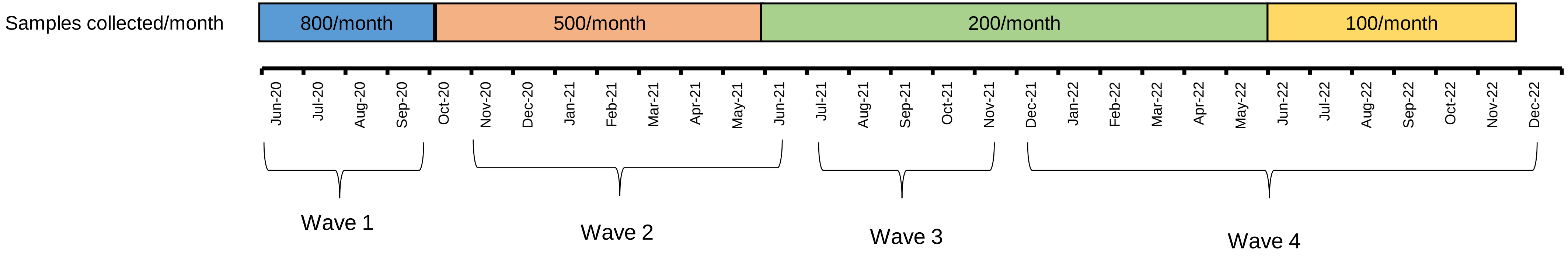

Supplement: Fig S1 — Sample collection strategy. [file spectrum.02625-24-s0001.pdf]
