# Supplementary figures and images for: Seroprevalence of SARS-CoV-2 IgG antibodies in children seeking medical care in Seattle, WA June 2020 to December 2022
Source: Microbiol Spectr. 2025 Mar 10;13(4):e02625-24. doi: 10.1128/spectrum.02625-24 (PMC11960482; doi:10.1128/spectrum.02625-24)

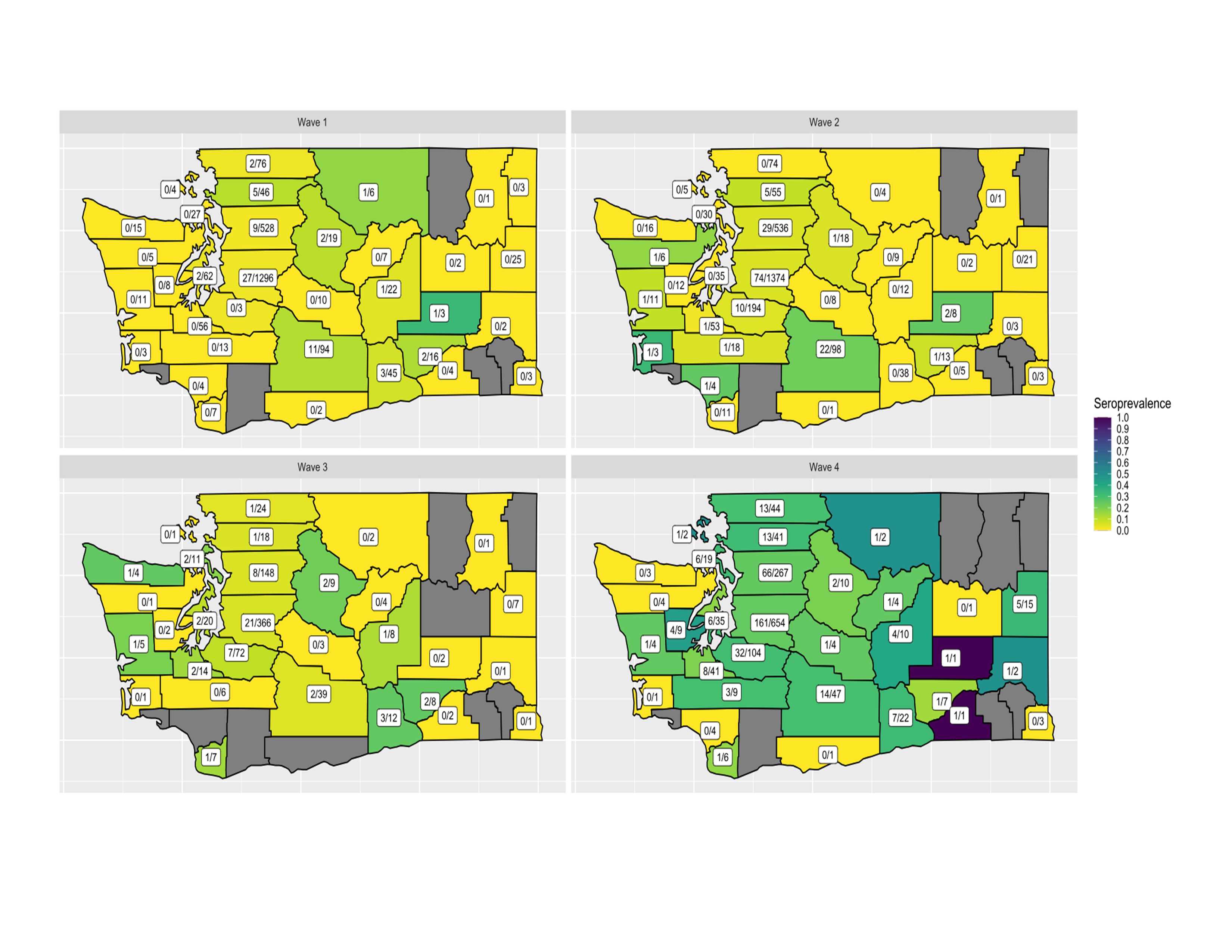

Supplement: Fig S2 — Seroprevalence by Washington State County by Wave. [file spectrum.02625-24-s0002.tif]

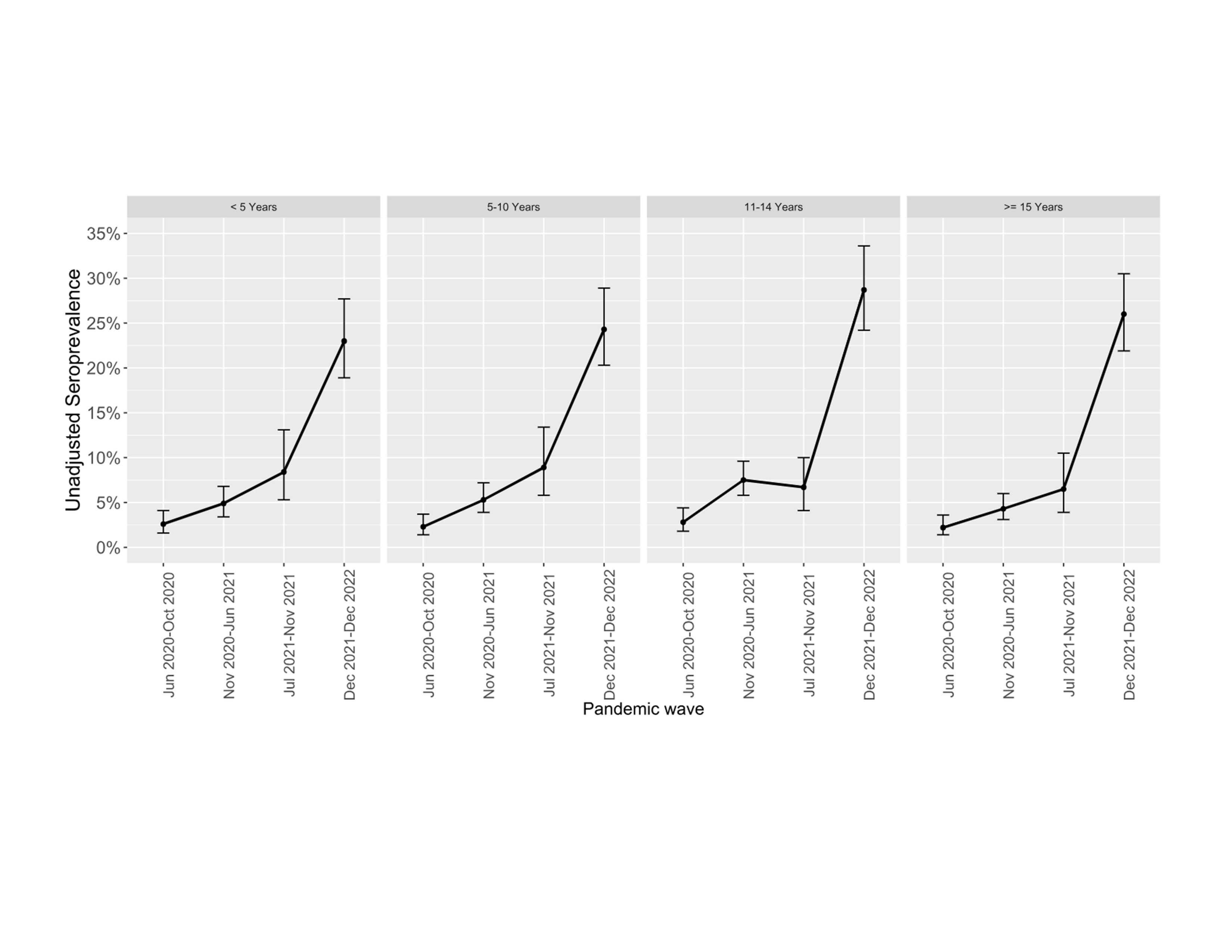

Supplement: Fig S3 — Seroprevalence by wave stratified by age group. [file spectrum.02625-24-s0003.tif]

**A**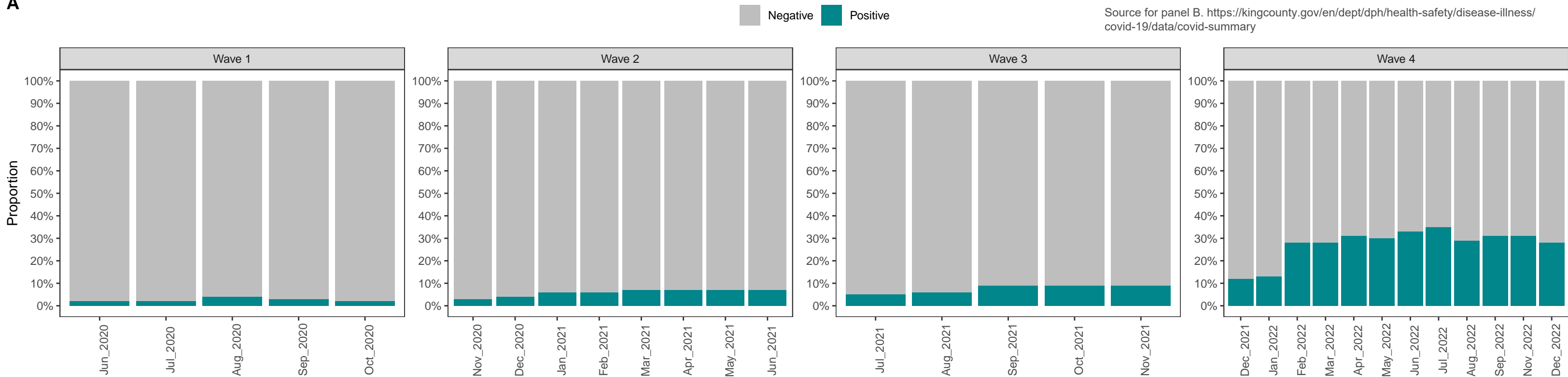**B**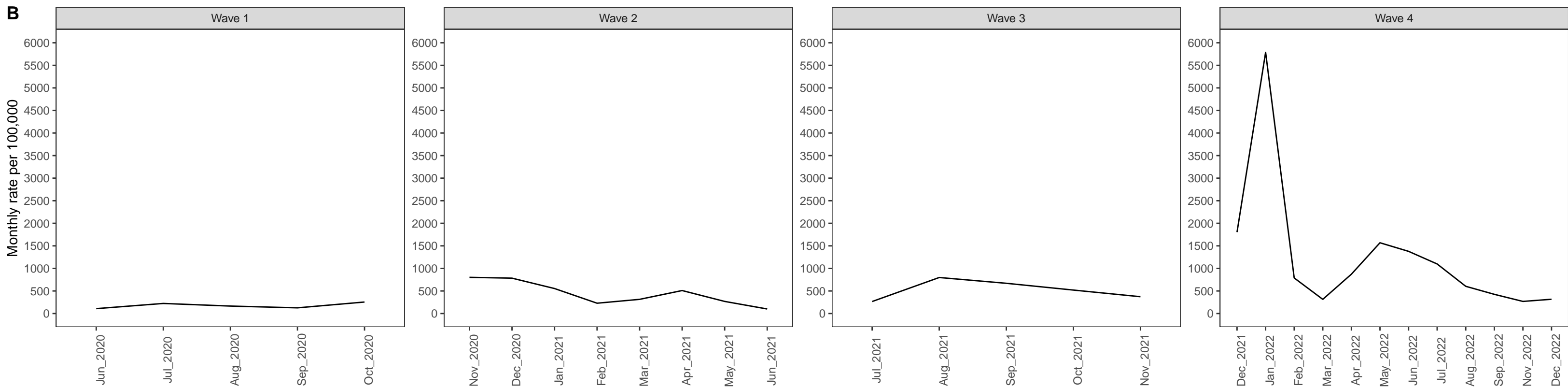

Supplement: Fig S4 — Proportion of positive samples and new case counts by month and wave. [file spectrum.02625-24-s0004.pdf]
