# Supplementary material for: Seroprevalence of SARS-CoV-2 IgG antibodies in children seeking medical care in Seattle, WA June 2020 to December 2022
Source: Microbiol Spectr. 2025 Mar 10;13(4):e02625-24. doi: 10.1128/spectrum.02625-24 (PMC11960482; doi:10.1128/spectrum.02625-24)
Supplement: Supplemental figure legends — Proportion of positive samples and new case counts by month and wave [file spectrum.02625-24-s0005.docx]

**Supp Figure 1.** Sample collection strategy and samples collected vs included in the analysis, by age group.

**Supp Figure 2.** Seroprevalence by Washington State County by Wave. Counties in gray represent those from which no samples were collected. The numbers indicate the number of seropositive samples over the total number of samples from children residing in that county.

**Supp Figure 3.** Seroprevalence by wave stratified by age group. Error bars indicate 95% confidence intervals.

**Supp Figure 4.** (A) Proportion of anti-N positive samples each collection month within each wave. (B) New case count per 100,000 King County residents each collection month within each wave.
